# Whole-Mount Immunostaining of Tyrosine Hydroxylase for Dopaminergic Neuron Analysis in Zebrafish Larvae

**DOI:** 10.21769/BioProtoc.5725

**Published:** 2026-07-05

**Authors:** Luís Félix

**Affiliations:** Centre for the Research and Technology of Agro-Environmental and Biological Sciences (CITAB), University of Trás-os-Montes and Alto Douro, Vila Real, Portugal

**Keywords:** Dopaminergic neurons, Zebrafish, Larvae, Whole-mount, Tyrosine hydroxylase

## Abstract

Whole-mount techniques are widely used in medical and biological research to analyze protein expression and tissue organization in intact specimens. Traditional approaches for protein localization include section-based immunohistochemistry and in situ hybridization; however, these methods can be limited by tissue disruption and loss of spatial context. Whole-mount protocols generally involve tissue fixation, permeabilization, and staining with specific probes, but their effectiveness varies depending on the antigen–antibody combination and the specimen type. Consequently, no universal protocol is suitable for all experimental conditions. This protocol presents a detailed whole-mount immunostaining protocol for evaluating tyrosine hydroxylase (TH) expression, a key marker of dopaminergic neurons, in zebrafish (*Danio rerio*) larvae. The procedure outlines critical steps from sample preparation to staining optimization to ensure reproducible and specific signal detection. This approach enables accurate visualization and analysis of dopaminergic neuron distribution in intact larvae. The protocol offers a reliable and adaptable approach that preserves tissue integrity, enables three-dimensional visualization, and is particularly well-suited for developmental and neurobiological studies using zebrafish larvae.

Key features

• Optimized whole-mount immunofluorescence protocol for detecting tyrosine hydroxylase in intact zebrafish larvae.

• Permeabilization and bleaching steps were included to improve antibody penetration and reduce pigmentation-related signal interference

• Three-dimensional brain architecture is preserved, enabling spatial analysis of dopaminergic neuron distribution without tissue sectioning, including region-specific fluorescence quantification using defined regions of interest (ROIs).

• Provides a straightforward approach for assessing dopaminergic neuron distribution through fluorescence imaging and quantitative analysis.

• Suitable for neurodevelopmental and neurotoxicity studies using zebrafish larvae.

## Graphical overview


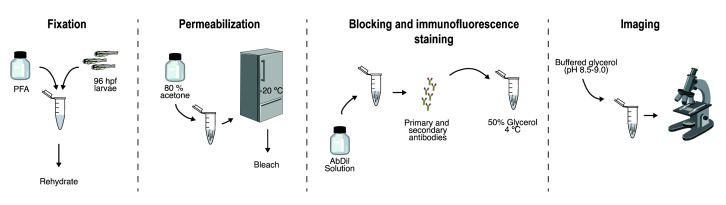


## Background

Whole-mount immunohistochemistry, a technique that combines the visualization of tissue structures with the specific detection of proteins using antibodies, can be traced back to the studies by Albert Coons and colleagues in 1941 [1]. Over the years, this technique has been widely used in many research laboratories (especially in developmental biology and neuroscience studies) as well as in clinical diagnostics [2,3]. In general, it can be applied to whole zygotic embryos as well as to dissected tissues, with fixation and preservation of tissue integrity [4], permeabilization to allow antibody access [5], and staining with specific probes [6,7] playing key roles in the successful application of this technique. Yet, despite significant developments and refinements, particularly in sample preparation and staining procedures, the selection of the most suitable method must be based on parameters such as the type of specimen under investigation and the degree of sensitivity required.

In different research areas, zebrafish are increasingly employed as model systems to investigate molecular, genetic, and cellular aspects of developmental and neurobiological processes due to several advantages over other animal models [8,9]. However, although zebrafish represent an excellent experimental system, protein detection methods must be carefully adjusted to account for factors such as tissue permeability, pigmentation, and developmental stage [10,11]. Indeed, there is no single protocol suitable for all experimental conditions, as the effective application of a specific working procedure depends strongly on the antigen–antibody combination [12,13].

Several alternative methodologies have been used to assess protein expression, including section-based immunohistochemistry, in situ hybridization, and tissue-clearing approaches coupled with advanced imaging techniques [14–16]. In addition, tissue- and cell-specific visualization in zebrafish is commonly achieved using transgenic reporter lines or chemical labeling techniques [17,18]. However, their use is limited by the availability of suitable promoters, variability in expression levels, and the need for specialized genetic tools and breeding strategies. Similarly, chemical approaches are often restricted to specific tissues or structures and may lack cellular resolution or specificity for particular protein targets. In contrast, whole-mount immunohistochemistry allows direct detection of endogenous proteins with cellular resolution, without the need for genetic modification. However, these techniques generally require specialized reagents, longer processing times, and access to advanced microscopy platforms. Therefore, in this chapter, a step-by-step protocol for the localization of tyrosine hydroxylase (TH), a standard marker of dopaminergic neurons in the central nervous system [19], in zebrafish larvae is presented. This protocol is adapted from previously published work [20] and includes critical steps and practical considerations to ensure reliable and reproducible staining. Compared with other published whole-mount approaches, the protocol described here emphasizes optimized permeabilization and bleaching steps to improve antibody penetration and minimize pigmentation-related background, making it particularly suitable for early larval stages. However, limitations include dependence on antibody quality and reduced applicability to later developmental stages or adult tissues without further protocol modification. Furthermore, despite recent advancements, the availability of validated antibodies in zebrafish remains limited, particularly for specific cell types or developmental stages, which can constrain immunohistochemical applications.

Beyond the assessment of dopaminergic neurons, this protocol can be readily adapted for the detection of additional neuronal or non-neuronal markers and applied in neurodevelopmental, neurotoxicity, and pharmacological studies using zebrafish larvae.

## Materials and reagents

**Biological materials**

1. 96 h post-fertilization (hpf) AB wild-type zebrafish larvae

**Reagents**

1. Sodium chloride (NaCl) (Sigma-Aldrich, catalog number: S7653)

2. Potassium chloride (KCl) (Sigma-Aldrich, catalog number: P3911)

3. Sodium phosphate dibasic (Na_2_HPO_4_) (Sigma-Aldrich, catalog number: S7907)

4. Potassium phosphate monobasic (KH_2_PO_4_) (Sigma-Aldrich, catalog number: P0662)

5. Sodium hydroxide (NaOH) (Sigma-Aldrich, catalog number: 221465)

6. Paraformaldehyde (PFA) (Sigma-Aldrich, catalog number: P6148)

7. Methanol (MeOH) (Sigma-Aldrich, catalog number: 34860)

8. Ethanol (EtOH) (Sigma-Aldrich, catalog number: E7023)

9. Acetone (Sigma-Aldrich, catalog number: 179124)

10. Triton X-100 (Sigma-Aldrich, catalog number: T8787)

11. Tween-20 (MP Biomedicals, catalog number: TWEEN201)

12. Potassium hydroxide (KOH) (Sigma-Aldrich, catalog number: 221473)

13. Hydrogen peroxide (H_2_O_2_) (Sigma-Aldrich, catalog number: 216763)

14. Bovine serum albumin (BSA) (Fisher Scientific, catalog number: BP9702)

15. Tyrosine hydroxylase antibody (Merck, catalog number: MAB318)

16. Alexa Fluor 488 secondary conjugated antibody (Jackson ImmunoResearch Europe Ltd., catalog number: 115-545-003)

17. Glycerol (Sigma-Aldrich, catalog number: 356352-M)

18. Sodium carbonate (Na_2_CO_3_) (Sigma-Aldrich, catalog number: S7795)

19. Sodium bicarbonate (NaHCO_3_) (Sigma-Aldrich, catalog number: S6297)

20. MilliQ water

21. Xylol (Sigma-Aldrich, catalog number: 1.08298)

**Solutions**

1. 10× phosphate-buffered saline (PBS) (see Recipes)

2. 1 N NaOH (see Recipes)

3. 4% PFA (see Recipes)

4. Ethanol (EtOH)/xylol solution (1:1 v/v) (see Recipes)

5. 80% acetone (see Recipes)

6. PTwx (see Recipes)

7. 100 mM KOH

8. 3% H_2_O_2_ (see Recipes)

9. Bleaching solution (see Recipes)

10. AbDil solution (see Recipes)

11. Glycerol solution (see Recipes)

12. 0.5 M carbonate buffer (pH 9.0) (see Recipes)

13. Buffered glycerol (see Recipes)

**Recipes**

**1. 10× PBS**

**Table:** 

Reagent	Final concentration	Quantity or volume
NaCl	1.37 M	40.0 g
KCl	27 mM	1.0 g
Na_2_HPO_4_	100 mM	8.9 g
KH_2_PO_4_	18 mM	1.2 g
MilliQ water		up to 500 mL
Total	n/a	500 mL

*Note: Adjust to neutral pH by adding acid or base as appropriate until the solution is pH 7.2–7.4. Sterilize by autoclaving for 20 min and store at 4 °C. Dilute in MilliQ water to 1*× *(v/v) before use.*

**2. 1 N NaOH**

**Table:** 

Reagent	Final concentration	Quantity or volume
NaOH	1 N	4.0 g
MilliQ water		100 mL
Total	n/a	100 mL

**3. 4% PFA**

**Table:** 

Reagent	Final concentration	Quantity or volume
Paraformaldehyde	4%	4.0 g
1× PBS		Up to 100 mL
Total	n/a	100 mL

*Note: Heat the solution to approximately 60 °C under stirring to facilitate dissolution. Avoid overheating and do not exceed 60 °C, as higher temperatures may lead to paraformaldehyde degradation. Slowly add 1 N NaOH until the solution clears. After, adjust pH to around 7.4 with diluted HCl and adjust the volume of the solution to 100 mL.*

**4. EtOH/xylol solution (1:1 v/v)**

**Table:** 

Reagent	Final concentration	Quantity or volume
Ethanol	50%	25.0 mL
Xylol	50%	25.0 mL
Total	n/a	50.0 mL

**5. 80% acetone**

**Table:** 

Reagent	Final concentration	Quantity or volume
Acetone	80%	80.0 mL
MilliQ water		20.0 mL
Total	n/a	100 mL

**6. PTwx**

**Table:** 

Reagent	Final concentration	Quantity or volume
Tween20	0.1%	100.0 μL
Triton X-100	0.1%	100.0 μL
PBS		99.8 mL
Total	n/a	100 mL

**7. 100 mM KOH**

**Table:** 

Reagent	Final concentration	Quantity or volume
KOH	100 mM	281 mg
MilliQ water		50.0 mL
Total	n/a	50.0 mL

**8. 3% H_2_O_2_**

**Table:** 

Reagent	Final concentration	Quantity or volume
H_2_O_2_	3%	5.0 mL
MilliQ water		45.0 mL
Total	n/a	50.0 mL

**9. Bleaching solution**

**Table:** 

Reagent	Final concentration	Quantity or volume
3% H_2_O_2_	1.5%	25.0 mL
100 mM KOH	50 mM	25.0 mL
Total	n/a	50.0 mL

**10. AbDil solution**

**Table:** 

Reagent	Final concentration	Quantity or volume
BSA	5%	0.5 g
Triton X-100	1%	100.0 μL
1× PBS		9.9 mL
Total	n/a	10.0 mL

**11. Glycerol solution**

**Table:** 

Reagent	Final concentration	Quantity or volume
Glycerol	50%–70%	50–70 mL
1× PBS		50–30 mL
Total	n/a	100 mL

**12. 0.5 M carbonate buffer (pH 9)**

**Table:** 

Reagent	Final concentration	Quantity or volume
Na_2_CO_3_	13 mM	0.138 g
NaHCO_3_	87 mM	0.731 g
NaCl	150 mM	0.877 g
MilliQ water		100.0 mL
Total	n/a	100 mL

**13. Buffered glycerol**

**Table:** 

Reagent	Final concentration	Quantity or volume
Glycerol		9 mL
0.5 M carbonate buffer	0.05 M	1 mL
Total	n/a	10 mL

**Laboratory supplies**

1. 1.5 mL microcentrifuge tubes

2. 2.0 mL microcentrifuge tubes

3. Routine glassware and consumables (e.g., pipette tips, conical tubes, etc.)

4. Glass microcapillaries (Merck, catalog number: P2049)

## Equipment

1. Rotator (e.g., Labnet Mini LabRoller Rotator)

2. Variable volume micropipettes

3. Stir plate

4. pH meter

5. Stereomicroscope (e.g., Olympus, model: SZX7)

6. Fluorescence microscope (e.g., Olympus, model: IX51 inverted fluorescence microscope)

## Software and datasets

1. Image analysis software (e.g., ImageJ [21])

2. Statistical software (e.g., GraphPad Prism [22])

## Procedure

**A. Zebrafish larvae collection**

1. AB wild-type zebrafish embryos are generated by pairwise mating using conventional methods [23].

2. Disinfect embryos [24] and sort them for viability at 2–3 h post-fertilization on a stereomicroscope.

3. Expose for 96 h to a selected drug/neurotoxin in E3 medium [25], under laboratory conditions [26], and according to standard guidelines [27].

**B. Larvae fixation**

1. At the end of the exposure period, collect 10 larvae in 1.5 mL centrifuge tubes and add 500 μL of 4% PFA.

2. Maintain at 4 °C for 14–18 h.

**Caution:** Fixation of zebrafish larvae in 4% PFA can be achieved at room temperature (RT) for 2–4 h. However, optimal tissue morphology has been achieved by overnight fixation at 4 °C. Over-fixation may lead to a higher background.

**Caution:** Fixed samples can be stored in 4% PFA at 4 °C for short periods (up to 24–48 h). Prolonged storage in PFA may lead to over-fixation and increased background fluorescence. Therefore, for longer storage, samples should be transferred to 100% methanol and kept at -20 °C.

**Caution:** Fixatives are broadly classified into aldehyde-based fixatives—such as formaldehyde, glutaraldehyde, and formalin—that work by creating bonds between lysine residues to cross-link sample proteins, and alcohol-based organic solvents—such as methanol, ethanol, and acetone—that work by dehydrating the sample material, which causes protein denaturation and precipitation. These latter fixatives do not require a separate permeabilization procedure. However, alcohol-based fixation can wash away soluble proteins and change the tertiary structure of target epitopes due to hydrophobic bond breaking. For this reason, aldehyde-based fixatives are preferred.

3. Wash three times in PBS for 5 min each at RT.

4. Store in 500 μL of 100% MeOH at -20 °C for at least 6 h.

**Critical:** Methanol is used to delipidate larvae. The larvae may be stored for several months and probably longer in this solution.

**Pause point:** Stop and continue the next day.

**C. Sample permeabilization and bleaching**

1. Replace MeOH by washing twice in 1 mL of EtOH.

2. Incubate samples in 1 mL of EtOH/xylol (1:1 v/v) for 1 h under rocking conditions.

**Caution:** Xylol is used as a rapid clearer for specimens that have been previously dehydrated in alcohol. However, as a relatively hazardous solvent, another xylene-free method can be adapted [28].

3. Wash twice in absolute ethanol for 5 min.

4. Rehydrate the sample for 15 min in 1 mL of descending ethanol concentrations (90%, 75%, 50%, 25%, and then H_2_O) containing 0.1% Tween 20.

5. Wash twice in H_2_O for 5 min.

6. Permeabilize the samples in 1 mL of 80% acetone at -20 °C for 40 min without shaking [29].

**Critical:** Acetone must be pre-chilled at -20 °C.

**Caution:** Acetone permeates the larvae, allowing the antibody to pass through the skin. Other permeabilizing reagents, such as Proteinase K or trypsin, can be used, and the amount used depends on the antibody of interest. However, acetone has been shown to be more effective than other permeabilization agents [20]. To aid in permeabilization, the skin of the larvae can be removed (by peeling it back with forceps).

7. Wash larvae twice in PTwx for 5 min.

8. Bleach larvae in bleaching solution for approximately 30 min or until pigmentation has completely disappeared.

**Critical:** Bleaching can damage tissue, so care must be taken to minimize the time larvae spend in the bleaching solution.

9. Wash larvae twice in PTwx for 5 min.

**Pause point:** If the protocol cannot be immediately continued, samples can be left in this solution for at least one week if stored refrigerated.

**D. Blocking and antibody incubation**

1. Block in AbDil solution under rocking conditions. Alternatively, blocking can be achieved by 2-h incubation at RT or overnight at 4 °C.

2. Rinse twice in PTwx.

3. Incubate in primary antibody (1:500) in AbDil overnight at 4 °C under rocking conditions.

**Critical:** The volume of antibody to be used should always be sufficient to cover the larvae, which should be between 100 and 500 μL per condition.

**Critical:** To achieve adequate immunostaining, shorter incubation times at 37 °C, longer incubation times (1–2 h) at room temperature, or extended overnight incubation at 4 °C can be used.

**Caution:** Antibodies can be reused. However, do not reuse more than twice. If reusing, store the removed primary antibody solution in an appropriate container at 4 °C.

4. Wash off excessive primary antibody 6 times for 5 min in PTwx.

5. Incubate in secondary antibody (1:500) in AbDil overnight at 4 °C on a shaker.

**Critical:** Secondary antibodies are photosensitive. Keep them protected from light as much as possible.

6. Wash three times in PTwx for 5 min each.

7. Rinse in PBS and store in 50% glycerol/PBS at 4 °C until imaging.

**E. Imaging and analysis**

1. Replace 50% glycerol/PBS with 70% glycerol/PBS and allow the larvae to sink to the bottom of the tube.

**Critical:** Before moving on to the next glycerol concentration, allow larvae to sink to the bottom of the tube, which can take up to 1 h.

**Critical:** Glycerol raises the refractive index of the mounting media, so brighter and higher resolution images will be obtained in a higher percentage of glycerol.

**Pause point:** Samples can be stored in 70% glycerol for up to one week at 4 °C. It is, however, strongly advised to image samples as soon as possible.

2. Replace 70% glycerol/PBS with buffered glycerol and store in the dark to preserve fluorescent signal.

*Note: Although other antifading agents could be used with higher antifading properties [30,31], glycerol is a common reagent in the laboratory and is easy to obtain.*

3. Aspirate the larvae into a microcapillary.

*Note: The use of a microcapillary facilitates precise handling and positioning of the larvae, allowing easy orientation of the specimen under the microscope and improving consistency during imaging.*

4. Image the samples using a fluorescence microscope with appropriate filter settings for the fluorophore used (e.g., FITC/Alexa Fluor 488).

**Critical:** Before imaging experimental groups, first acquire images from control samples to define optimal microscope settings (e.g., exposure time, gain, and illumination). Once established, these settings should be kept constant for all subsequent samples to ensure comparability between conditions.

**Figure 1.**
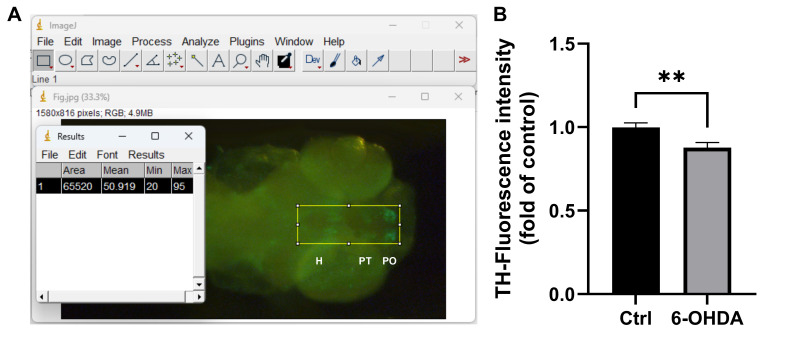
Main steps in conducting fluorescence analysis from the whole-mount assay in zebrafish larvae. (A) Fluorescence analysis using the ImageJ software. A representative image of zebrafish larvae with fluorescent signals is shown. In addition, an ImageJ window displaying the ROI and the fluorescence intensity is shown. Dopaminergic neurons in zebrafish larvae are primarily organized in clusters within the preoptic area (PO), posterior tuberculum (PT), and hypothalamus (H) [32]. (B) Quantification of tyrosine hydroxylase (TH) in zebrafish larvae exposed to a neurotoxin, 6-OHDA. Data is expressed as mean and standard deviation of five independent replicates, normalized to the control values. Asterisks (**) indicate significant differences when compared to the control (Ctrl) group (t-test, p < 0.01).

5. Acquire images focusing on the brain regions containing dopaminergic neurons.

6. After imaging, use ImageJ to analyze the data. Open ImageJ and draw a region of interest (ROI) in the brain of zebrafish larvae (Figure 1). Go to *Analyse* > *Set Measurements* to configure the desired parameters and check if *Area* and *Mean Grey Value* are selected. Go to *Analyse* > *Measure*, and a pop-up window will appear with your measurements. Copy the value under *Mean*, which corresponds to the mean fluorescence of your ROI, to a spreadsheet. Move the ROI to the background, repeat the measurement, and correct the fluorescence obtained.

## Data analysis

Fluorescence images were analyzed using ImageJ software. For each zebrafish larva, a region of interest (ROI) was manually defined in the brain area containing dopaminergic neurons. Mean fluorescence intensity was measured for each ROI after background subtraction by placing an equivalent ROI in an adjacent nonfluorescent area. For each experimental condition, five independent biological replicates (each containing 10 individual larvae) were analyzed. One ROI per larva was quantified, and the average of the 10 animals was measured as n = 1. Larvae presenting tissue damage, poor antibody penetration, or imaging artifacts were excluded from the analysis. No additional outlier removal was performed. Fluorescence intensity values were normalized to the control group and expressed as mean ± standard deviation. Statistical analyses were performed using GraphPad Prism version 9.1 (GraphPad Software, USA). Comparisons between treated and control groups were conducted using Student’s t-test. Differences were considered statistically significant when p < 0.05. No advanced computational or programming skills are required to perform the data analysis.

## Validation of protocol

This protocol (or parts of it) has been used and validated in the following research article(s):

• Gomes et al. [36]. Protective effects of 24-epibrassinolide against the 6-OHDA zebrafish model of Parkinson's disease. *Comparative Biochemistry and Physiology Part C: Toxicology & Pharmacology* (Figure 3 and Figure S1). https://doi.org/10.1016/j.cbpc.2023.109630

• Carneiro et al. [37]. Amelioration of 6-OHDA-induced Parkinson’s symptoms in zebrafish larvae by an almond skin acetonic extract. *International Journal of Molecular Sciences* (Figure 2). https://doi.org/10.3390/ijms27062590

## General notes and troubleshooting

This protocol provides a robust and reproducible approach for whole-mount immunostaining of zebrafish larvae, with particular suitability for the detection of dopaminergic neurons via tyrosine hydroxylase (TH) analysis. However, several factors may influence the outcome and should be considered. For instance, the antibody quality and specificity, which may vary between batches and suppliers, can result in different outcomes. Optimization of antibody concentration and incubation conditions may be required when adapting the protocol to different targets. In addition, the efficiency of antibody penetration is highly dependent on proper permeabilization, as insufficient permeabilization may result in weak or uneven staining, while excessive treatment can damage tissue integrity.

Pigmentation in zebrafish larvae can also interfere with signal detection. Although the bleaching step reduces this issue, prolonged exposure may compromise tissue morphology. Therefore, careful monitoring of bleaching duration is recommended. Notably, this protocol does not require the use of phenylthiourea (PTU) to inhibit pigmentation, thereby avoiding potential developmental effects associated with PTU treatment [33–35].

In addition, this protocol is optimized for early larval stages, and its direct application to later developmental stages or adult tissues may require further adjustments, particularly in permeabilization and clearing steps. Furthermore, variability in imaging conditions, including microscope settings and sample orientation, may affect fluorescence quantification. To ensure consistency, it is recommended to standardize imaging parameters and maintain consistent positioning of larvae across samples.

Despite these considerations, the protocol can be adapted to detect other neuronal or non-neuronal markers, provided that appropriate optimization of antibody conditions is performed.
